# Effects of Anti-IL-17 on Inflammation, Remodeling, and Oxidative Stress in an Experimental Model of Asthma Exacerbated by LPS

**DOI:** 10.3389/fimmu.2017.01835

**Published:** 2018-01-05

**Authors:** Leandro do Nascimento Camargo, Renato Fraga Righetti, Luciana Ritha de Cássia Rolim Barbosa Aristóteles, Tabata Maruyama dos Santos, Flávia Castro Ribas de Souza, Silvia Fukuzaki, Maysa Mariana Cruz, Maria Isabel Cardoso Alonso-Vale, Beatriz Mangueira Saraiva-Romanholo, Carla Máximo Prado, Mílton de Arruda Martins, Edna Aparecida Leick, Iolanda de Fátima Lopes Calvo Tibério

**Affiliations:** ^1^Department of Medical Sciences, School of Medicine, University of São Paulo, São Paulo, Brazil; ^2^Hospital Sírio-Libanês, São Paulo, Brazil; ^3^Department of Biological Sciences, Institute of Biomedical Sciences, Federal University of São Paulo, São Paulo, Brazil; ^4^Department of Biological Sciences, Federal University of São Paulo, São Paulo, Brazil

**Keywords:** anti-IL-17, distal lung, asthma, inflammation, LPS-exacerbated

## Abstract

Inflammation plays a central role in the development of asthma, which is considered an allergic disease with a classic Th2 inflammatory profile. However, cytokine IL-17 has been examined to better understand the pathophysiology of this disease. Severe asthmatic patients experience frequent exacerbations, leading to infection, and subsequently show altered levels of inflammation that are unlikely to be due to the Th2 immune response alone. This study estimates the effects of anti-IL-17 therapy in the pulmonary parenchyma in a murine asthma model exacerbated by LPS. BALB/c mice were sensitized with intraperitoneal ovalbumin and repeatedly exposed to inhalation with ovalbumin, followed by treatment with or without anti-IL-17. Twenty-four hours prior to the end of the 29-day experimental protocol, the two groups received LPS (0.1 mg/ml intratracheal OVA-LPS and OVA-LPS IL-17). We subsequently evaluated bronchoalveolar lavage fluid, performed a lung tissue morphometric analysis, and measured IL-6 gene expression. OVA-LPS-treated animals treated with anti-IL-17 showed decreased pulmonary inflammation, edema, oxidative stress, and extracellular matrix remodeling compared to the non-treated OVA and OVA-LPS groups (*p* < 0.05). The anti-IL-17 treatment also decreased the numbers of dendritic cells, FOXP3, NF-κB, and Rho kinase 1- and 2-positive cells compared to the non-treated OVA and OVA-LPS groups (*p* < 0.05). In conclusion, these data suggest that inhibition of IL-17 is a promising therapeutic avenue, even in exacerbated asthmatic patients, and significantly contributes to the control of Th1/Th2/Th17 inflammation, chemokine expression, extracellular matrix remodeling, and oxidative stress in a murine experimental asthma model exacerbated by LPS.

## Introduction

Bronchial asthma is an inflammatory, obstructive, heterogeneous, complex, and chronic lung disease characterized by an increase in mucus secretion and hyperreactivity of the airways. Over time, functional and structural changes in lung tissue may occur (1). Bronchial asthma is defined based on clinical and pathophysiological characteristics. Patients with a pre-existing diagnosis or even an initial presentation of asthma can have exacerbations (2).

LPS is a glycolipid that is present in the outer membrane of Gram-negative bacteria and is an important activator of the innate immune response *via* toll-like receptor 4. It has little toxicity when used directly to treat cells (*in vitro*). The use of this endotoxin provides information on the effects of the inflammatory response by bacterial infection (3). Starkhammar et al. (4) used an experimental model with a low dose of LPS (0.1 mg/ml) and found increased airway hyperresponsiveness and neutrophil-dominant inflammation, which is common in asthmatic patients with exacerbations caused by bacteria. However, the use of LPS in high doses reproduces a experimental model of acute lung injury (5).

Th17 cells act as potent inducers of inflammatory responses as a determining factor for severe asthma (6). In addition to displaying an elevated anti-inflammatory profile, increased IL-17A and IL-17F levels may aggravate the neutrophil inflammatory response (7). IL-17 promotes eosinophilia in airways through chemokine growth factors (G-CSF, GM-CSF, and TNF-α) (8). In addition, IL-17 may contribute to the remodeling of airways in asthma, suggesting that blockade of this cytokine may facilitate fibrosis control (9). Furthermore, allergic asthma may be influenced by the IL-17 levels, which directly increase smooth muscle cells in airways *via* the NF-κB signaling pathway. NF-κB modulates expression of the GTPase RhoA and its effector kinases ROCK-1 and ROCK-2, which are signaling factors that are critical for airway smooth muscle contraction (10). Thus, the NF-κB, Rho kinase, and IL-17 pathways may be attractive therapeutic targets for the treatment of AHR and inflammation (11).

Although previous studies have demonstrated the role of IL-17 in hyperreactivity (4, 12), few studies have evaluated the effects of LPS in animal models allergic inflammation. Lowe et al. (13) showed the effects of exposure to LPS and OVA in mice treated with corticosteroids. They observed changes in the neutrophils, eosinophils, and functional inflammatory responses, and these effects prolonged asthmatic responses and decreased corticosteroid sensitivity. Bae et al. (14) confirmed that the exacerbations are associated to of neutrophilic responses and increase in IL-17 levels due to LPS treatment in asthma model. However, there are no studies evaluating the chemokines and signaling pathways involved in allergic inflammation exacerbated by LPS. In addition, remodeling of the extracellular matrix and oxidative stress may be potentiated in bacterial infections. Although asthma is considered to be an airway disease, this condition has long been characterized by the contribution of distal parenchymal changes in asthmatic responses, and the development of alterations in the distal lungs of asthmatic patients has an important impact on the pathogenesis and treatment of this disease (15, 16). Thus, the pulmonary parenchyma may play a fundamental role in total pulmonary resistance in addition to its potential participation in the pathophysiology of asthma, which may alter the potential treatment of this disease, particularly in severe asthma (17–19).

We recently demonstrated the presence of hyperresponsiveness, an increase in inflammatory cells, activation of the oxidative stress pathway and remodeling of the extracellular matrix in the distal parenchyma of chronic allergic inflammation models (10, 20).

Although IL-17 shows potential as an alternative for asthma treatment, there are no studies evaluating the use of anti-IL-17 in LPS-exacerbated asthma models. The aim of this study was to examine the levels of oxidative stress, inflammation, and extracellular remodeling in the lung parenchyma of an animal model of LPS-exacerbated asthma.

## Materials and Methods

### Experimental Groups

The present study was approved by the Research Ethics Committee of the Hospital das Clínicas of the Faculty of Medicine of the University of São Paulo (Case No. 141/16).

This protocol was repeated twice and used a total of 60 animals male BALB/c mice from the Medical School at the University of São Paulo were utilized in accordance with the Guideline to Care and Use of Laboratory Animals, published by the National Institutes of Health (21) (NIH publication 85-23, revised in 1985). On average, the body weight of the animals was approximately 20–25 g at the beginning of the sensitization protocol. The animals were divided into the following groups: the SAL group, which received inhalations with a sterile saline solution (*n* = 6); the OVA group, which received inhalations of an ovalbumin solution (*n* = 6); the OVA anti-IL-17 group, which received inhalations of an OVA solution and treatment with an anti-IL-17 monoclonal antibody (*n* = 6); the OVA-LPS group, which received inhalations of an ovalbumin solution and LPS instillation (*n* = 6); and the OVA-LPS anti-IL-17 group, which received inhalations of an ovalbumin solution, LPS instillation and treatment with an anti-IL-17 monoclonal antibody (*n* = 6).

### Sensitization Protocol

**Figure 1:**
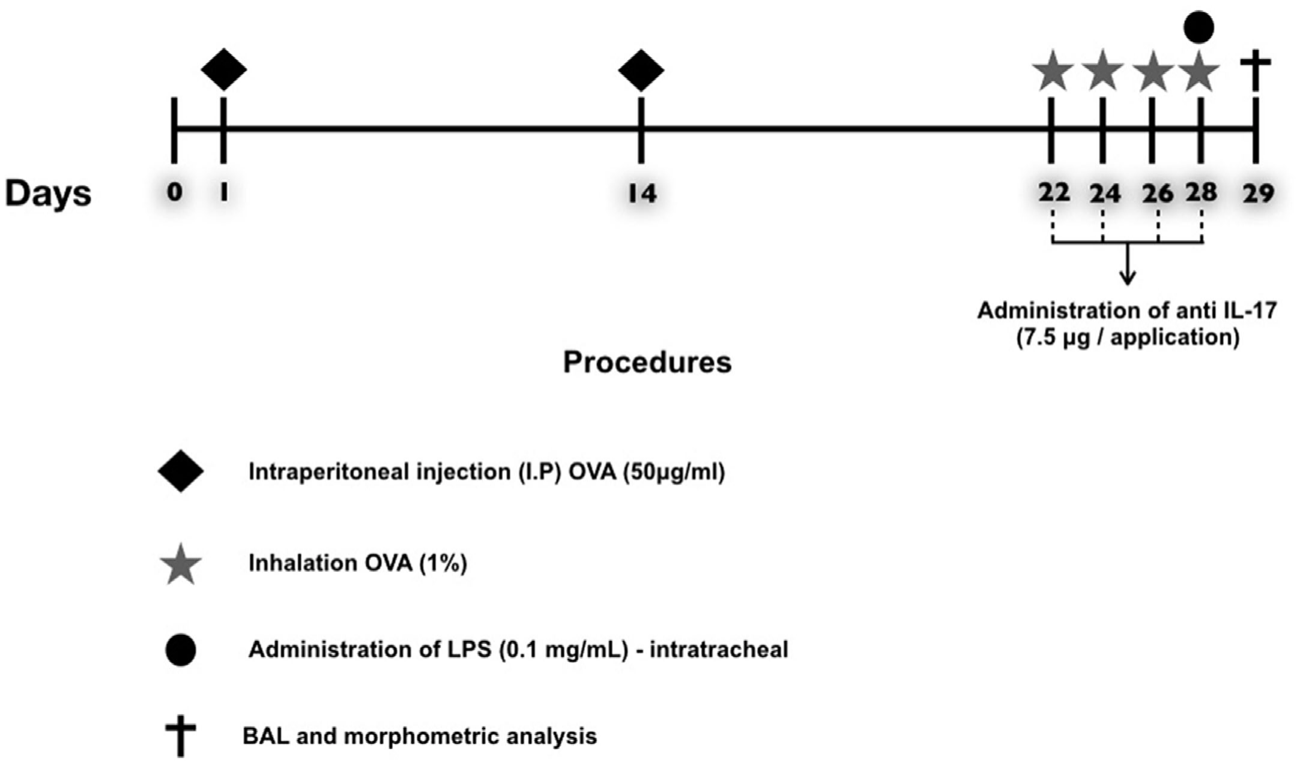
Timeline of the protocol for establishing chronic allergic inflammation + exacerbation. On days 1 and 14, the OVA, OVA-LPS, OVA anti-IL-17, and OVA-LPS anti-IL-17 groups were sensitized with OVA (i.p.) and the SAL control group received saline (i.p.). On days 22, 24, 26, and 28, 1 h prior to inhalation challenge, the treatment groups received anti-IL-17 (i.p.), and 24 h after the end of the protocol, the OVA-LPS and OVA-LPS anti-IL-17 groups received intratracheal LPS.

A schematic describing the 29-day ovalbumin sensitization protocol used in this study is shown in Figure 1. Mice received a solution of 50 µg of ovalbumin (*GRADE IV*, Sigma-Aldrich, St. Louis, MO, USA) and 6 mg of aluminum hydroxide (Alumen; Pepsamar, Sanofi-Synthelabo S.A., Rio de Janeiro, Brazil) in a total volume of 0.2 ml *via* intraperitoneal injection on days 1 and 14. The animals inhaled an OVA aerosol diluted in 0.9% NaCl (physiological saline) at a final concentration of 10 mg/ml (1%) on days 22, 24, 26, and 28. In addition, the SAL group received a saline solution (NaCl 0.9%) and aluminum hydroxide (Alumen) (6 mg) *via* intraperitoneal injection; on the inhalation challenge days, the mice were exposed to 0.9% saline solution through aerosol for 30 min (22). The LPS administration was based on the protocol of Starkhammar et al. (4), who used a dose that only caused hyperresponsiveness and neutrophil recruitment. The treatment was performed with 20 µl of phosphate-buffered saline (PBS) + 0.1 mg/ml *Escherichia coli* 0127: B8 (Sigma-Aldrich, St. Louis, MO, USA) 24 h after the last antigen challenge on day 29. The animals in the LPS-OVA and anti-LPS-OVA IL-17 groups were anesthetized *via* inhalation of isoflurane and received LPS intratracheally. No animal died during this protocol.

### Treatment

An anti-IL-17A (clone50104) neutralizing antibody (R and D Systems, Abingdon, UK) was administered *via* intraperitoneal injection 1 h prior to each aerosol administration on days 22, 24, 26, and 28 of the experimental asthma protocol and 1 h before LPS administration, at a dose of 7.5 μg/application, based on the Barlow’s protocol (23).

### Bronchoalveolar Lavage Fluid (BALF)

Animals were anesthetized with thiopental (250 mg/kg i.p.) 24 h after the end of the protocol. The anterior chest wall was opened, animals were exsanguinated *via* the abdominal aorta, and BALF was collected. The trachea was cannulated, and BALF was obtained by washing the airway lumina with 3× 0.5 ml of sterile saline; for each 0.5 ml instilled, the volume was recovered (totaling 1.5 ml). The BALF sample was centrifuged at 790 *g* for 10 min at 5°C. The cell pellet was resuspended in 300 µl of saline. Subsequently, after vortex mixing, 100 µl of this sample was used for lamina preparation for differential counting. The remaining BALF was cytocentrifuged in a Cytospin for 6 min at 450 rpm, and the lamina was subsequently stained with *DiffQuick*. The total cell count was obtained by optical microscopy and a *Neubauer* hemocytometer (400× magnification). The differentiation of neutrophils, eosinophils, lymphocytes, and macrophages was performed using an optical microscope with an immersion objective lens (1,000× magnification). At the end of this procedure, the lungs were isolated and removed for histological analysis.

### Mean Alveolar Diameter (Lm)

The Lm was measured using a microscope (E200MV, Nikon Corporation, Tokyo, Japan) with a 400× magnification and a reticulum with a known area (50 straight and 100 points) to calculate how many times the reticular lines intercepted the alveolar walls. The initial area of the alveolar walls was determined, excluding vessels and airways. For each animal, 20 lung parenchyma fields were randomly analyzed. Thus, the mean alveolar diameter was calculated according to the ratio of the area of the pulmonary parenchyma to the number of intersections between the lines and the alveolar walls. All Lm values were expressed in micrometers (24). We used this method of analysis to rule out any acute lung injury at the dose of LPS used in the present protocol.

### Immunohistochemistry

**Figure 2:**
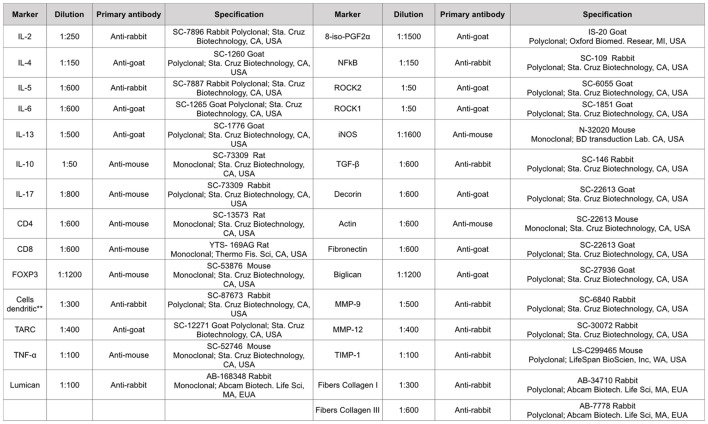
Types of antibodies and dilutions used in the study. **This monoclonal antibody is per DC-STAMP that represents a multi-membrane spanning protein preferentially expressed for dendritic cells. DC-STAMP is present in the endoplasmic reticulum of immature DC’s and it can translocate to the Golgi compartment during maturation.

Pulmonary tissue fragments were fixed in 10% formalin and embedded in paraffin. Five-micrometer thick sections were stained with hematoxylin and eosin for further analysis. The material was subjected to standard histological techniques as described below. The slices were deparaffinized and rehydrated for immunohistochemistry, treated with Proteinase K for 20 min at 37°C followed by 20 min at room temperature, and washed with PBS. Blocking of endogenous peroxidases was performed by incubation with 3% hydrogen peroxide (H_2_O_2_) 10 V (3× 10 min) and sections of experimental and control (positive and negative) tissue slides were incubated overnight with the indicated antibodies (Figure 2). The following day, the slides were washed in PBS and incubated with a secondary antibody using ABCKit by Vectastain (Vector Elite-PK-6105 anti-goat), PK-6101 (anti-rabbit), and PK-6102 (anti-mouse). For visualization of positive cells, the slides were washed in PBS and proteins were visualized using 3,3’-diaminobenzidine chromosome (DAB) (Sigma Chemical Co., St. Louis, MO, USA). Slide sections were contrasted with Harris hematoxylin (Merck, Darmstadt, Germany) and assembled using Entellan microscopy resin (Merck) (25). Analyzes were performed using the morphometric technique described below.

### Morphometric Analysis for Inflammatory and Edema Evaluation

The point-counting technique was employed (26) by means of a reticule of a known area (50 lines and 100 points) coupled to a microscope (E200MV, Nikon Corporation, Tokyo, Japan). Ten random fields in the alveolar walls per animal, at a magnification of 1,000×, were counted, and the number of positive cells was established based on the number of points that coincided with the positive cells within the reticulum divided by the number of points coinciding with the alveolar walls. The total area of the reticulum was 10^4^ μm^2^ and analyzes were performed at 1,000× magnification (17, 27).

The optical density was used to evaluate the volume fractions of the collagen fibers type I and III, actin, decorin, lumican, biglycan, fibronectin, and isoprostane PGF2α. Images were captured using an Leica DM2500 microscope (Leica Microsystems, Wetzlar, Germany), a digital camera (Leica DFC420 Leica Microsystems, Wetzlar, Germany). The images were acquired and processed using Optimas v.4.10 software. We analyzed 10 fields per lamina and one lamina per animal. The images were analyzed using Image-Proplus 4.5 software (NIH, MD, USA). This software allowed a thresholding of the color shades to be developed. These shades represent the positive areas quantified in the previously determined area. The volume fractions of these markers are expressed as percentages of the area (20).

By using a light microscope (Leica DFC 420 Leica microsystems, Wetzlar, Germany), eight non-overlapping fields of view were imaged at 100×, 200×, and 400× magnifications. We used a weighted scoring system to quantify interstitial edema. Scores from 0 to 4 were used to represent the severity of interstitial edema, with 0 representing no effect and 4 representing maximal severity. Additionally, the degree of each score quantified per field of view was established on a scale of 0–4, with 0 representing no noticeable extent and 4 representing complete involvement. An evaluator blind to all animal treatment conditions performed all analyzes and product of severity and the extent of each feature, ranging from 0 to 16, was used to calculate the overall scores (28, 29).

### Gene Expression

The expression level of IL-6R in the lungs was evaluated using real-time PCR (polymerase chain reaction) as previously reported by our group (30). Expression of GAPDH was used as an internal control. The primer sequences and annealing temperatures were: GAPDH (5′–3′ sense: CCACCACCCTGTTGCTGTAG; 5′–3′antisense: CTTGGGCTACACTGAGGACC; 60°C; NM_008084) and IL-6R (5′–3′ sense: TTCTCTGGGAAATCGTGGAAA; 5′–3′ antisense: TCAGAATTGCCATTGCACAAC; 60°C; NM_001314054). The results were obtained as cycle number at which logarithmic PCR plots cross a calculated threshold line and used to determine ΔCt values [ΔCt = (Ct of the target gene) − (Ct of the house-keeping gene)]. The results were expressed as arbitrary units using the transformation: Expression = 1000 × (2^−Δct^) arbitrary units.

### Statistical Analysis

All data are represented as the means ± SEs, and graphs are presented in bar formats. One-way Analysis of Variance (ANOVA) followed by the *Holm-Sidak* method for multiple comparisons was used to determine the difference between groups with statistical significance. All analyzes were conducted using SigmaPlot 11.0 software (Systat Software, SPSS Inc., USA). A *p*-value < 0.05 was considered statistically significant.

## Results

### Mean Linear Intercept (Lm)

The mean linear values of the alveolar intercepts of the experimental groups were as follows: Group OVA (40.82 ± 1.55), OVA-LPS (44.40 ± 0.58), and SAL (42.23 ± 0.46), which were not significantly different. The OVA group treated with anti-IL-17 (41.93 ± 0.60) and the OVA-LPS group treated with anti-IL-17 (42.29 ± 0.51) had no significant differences compared to the OVA and OVA-LPS groups.

### Effects of Anti-IL-17 on BALF

**Figure 3:**
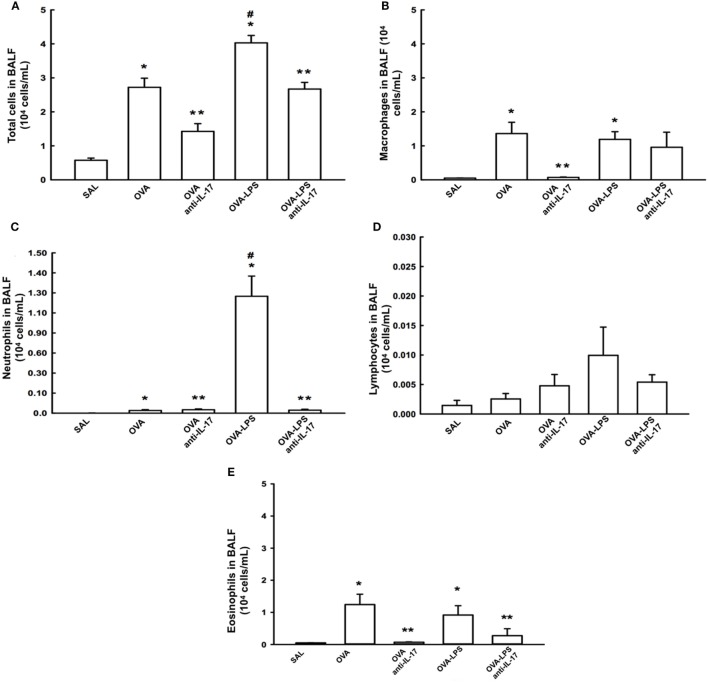
Effects of anti IL-17 treatment on bronchoalveolar lavage fluid (BALF) **(A–E)**. **(A)** Total cells, **(B)** cell differential for macrophages, **(C)** cell differential for neutrophils, **(D)** cell differential for Lymphocytes, and **(E)** cell differential for eosinophils. The results are expressed as 10^4^ cells/ml. Data are presented as the means ± SEs. The differences were considered significant when *p* < 0.05. *p* < 0.05 vs. SAL group; **p < 0.05 vs. OVA and OVA-LPS groups. ^#^*p* < 0.05 vs. OVA group.

The total cell count in the BALF and the differential count for macrophages, lymphocytes, neutrophils, and eosinophils are shown in Figures 3A–E The evaluation of the total and the differential cell counts for macrophages, neutrophils, and eosinophils revealed an increase of type cells in the OVA and OVA-LPS groups compared to the SAL group (*p* < 0.05). The OVA-LPS group showed an increase in the number of total cells and neutrophils compared to the OVA group (*p* < 0.05). The animals from the OVA anti-IL-17 and OVA-LPS anti-IL-17 groups showed decreases in the numbers of total cells, macrophages, and neutrophils compared to the OVA and OVA-LPS groups, respectively (*p* < 0.05). However, eosinophils decreased only in the OVA anti-IL-17 group compared to the OVA group (*p* < 0.05). There was no significant difference in the counts of lymphocytes in any of the groups. The following is the description of the cell differences in percentage for: 1. Macrophages: SAL (8.76 ± 0.35%), OVA (48.15 ± 7.94%), OVA-anti-IL-17 (5.57 ± 0.90%), OVA-LPS (33.78 ± 3.00%), and OVA-LPS anti-IL-17 (14.29 ± 7.33%) groups; 2. Neutrophils: SAL (0.20 ± 0.03%), OVA (0.75 ± 0.29%), OVA-anti-IL-17 (2.31 ± 0.43%), OVA-LPS (30.14 ± 5.72%), and OVA-LPS anti-IL-17 (1.05 ± 0.32%) groups; 3. Lymphocytes: SAL (0.10 ± 0.04%), OVA (0.16 ± 0.04%), OVA-anti-IL-17 (0.24 ± 0.14%), OVA-LPS (0.40 ± 0.18%), and OVA-LPS-anti-IL-17 (0.21 ± 0.12%) groups; 4. Eosinophils: SAL (0.24 ± 0.0%), OVA (67.14 ± 31.76%), OVA-anti-17 (1.23 ± 0.10%), OVA-LPS (28.95 ± 1.58%), and OVA-LPS anti-IL-17 (8.91 ± 8.38%) groups.

### Effect of Anti-IL-17 on Gene and Cell Expression of IL-6

**Figure 4:**
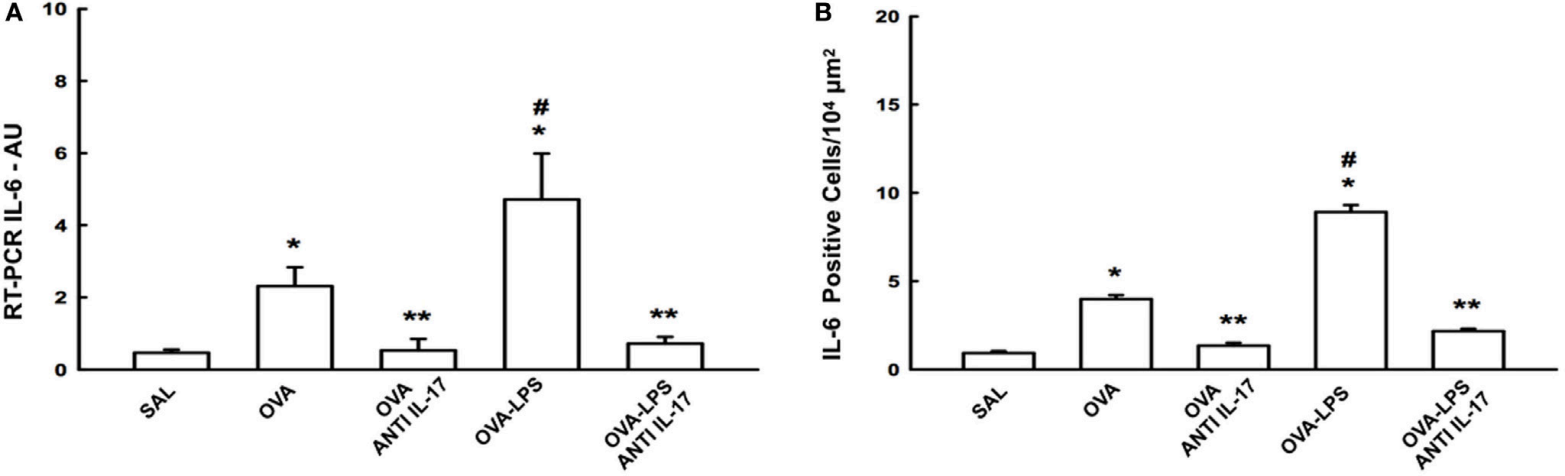
Effects of anti-IL-17 on IL-6 gene expression and IL-6 positive cell number. **(A)** The levels of IL-6 mRNA in the lung parenchyma were evaluated using RT-PCR (AU) and **(B)** IL-6-positive cells. The results were expressed as arbitrary units and as positive cells 10^4^ µm^2^. Data represent means ± SEs. The differences were considered significant when *p* < 0.05. *p < 0.05 vs. SAL group; **p < 0.05 vs. OVA and OVA-LPS groups. ^#^*p* < 0.05 vs. OVA group.

We compared the results of IL-6 gene expression analysis by PCR with the cell expression of this cytokine assessed by morphometric analysis following immunohistochemical staining. The number of positive cells and gene expression of IL-6 in the lung parenchyma is presented in Figure 4A. There was an increase in the number of positive cells in the lung parenchyma in the OVA and OVA-LPS groups compared to the SAL group (*p* < 0.05). The OVA-LPS group showed an increase in relation to the OVA group (*p* < 0.05). Treatment with anti-IL-17 attenuated all inflammatory markers evaluated in the OVA anti-IL-17 and OVA-LPS anti-IL-17 groups compared to the OVA and OVA-LPS groups (*p* < 0.05). The gene expression evaluation by PCR showed similar patterns of responses compared to morphometric analyzes, as shown in Figure 4B. Considering these results, we decided to evaluate additional inflammatory, remodeling, and oxidative stress markers using morphometric analysis after immunohistochemistry studies.

### Effects of Anti-IL-17 Therapy on Inflammation

**Figure 5:**
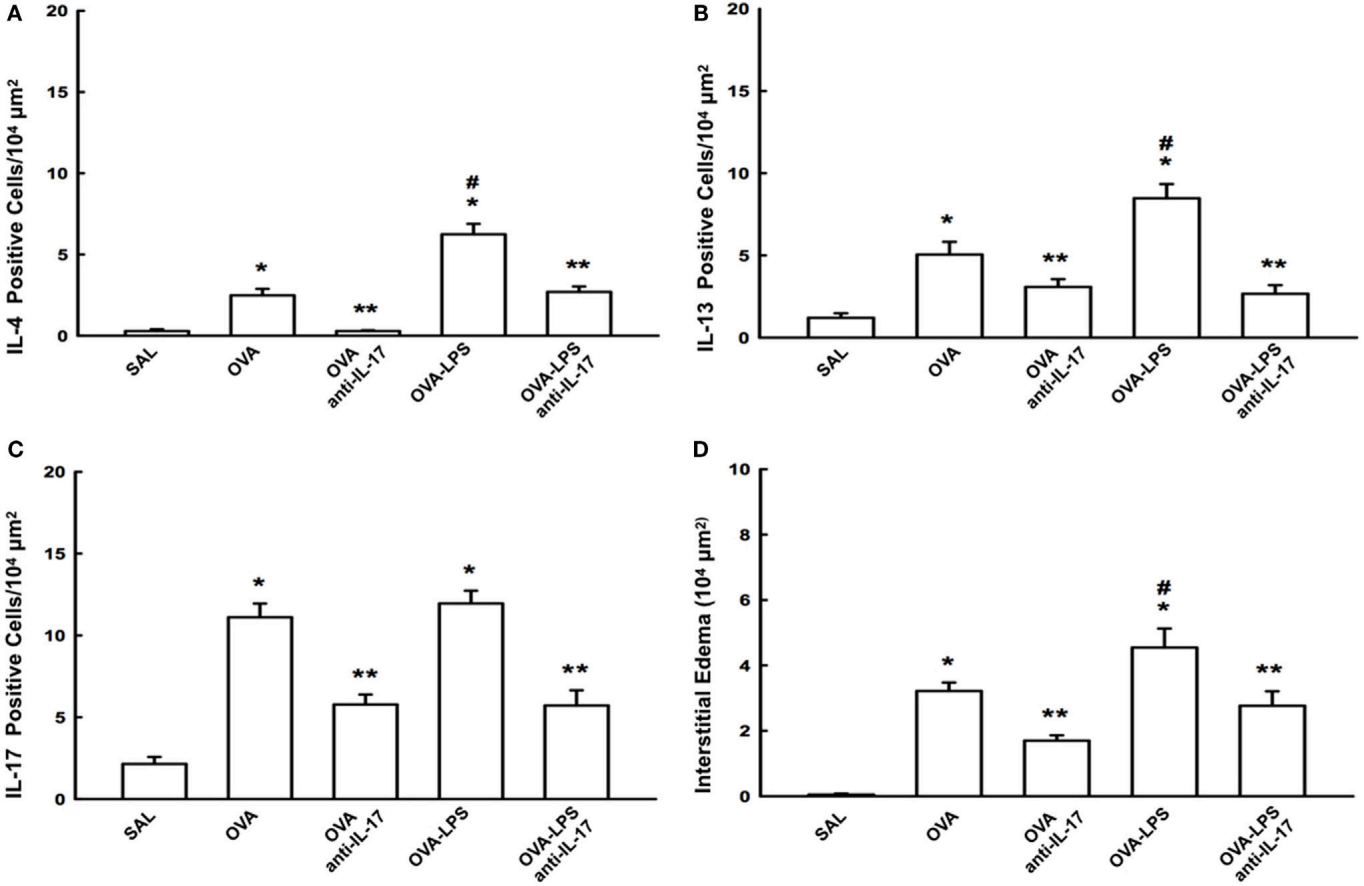
Effects of anti-IL-17 on inflammatory cells and interstitial edema of the pulmonary parenchyma **(A–D)**. **(A)** IL-4, **(B)** IL-13, and **(C)** IL-17 levels expressed as numbers of positive cells/10^4^ µm^2^, and **(D)** interstitial edema area in 10^4^ µm^2^. Data are presented as the means ± SEs. The differences were considered significant when *p* < 0.05 (*p* < 0.05 vs. SAL group; ** *p* < 0.05 vs. OVA and OVA-LPS groups; ^#^*p* < 0.05 vs. OVA group).

The number of cells positive for IL-4, IL-13, IL-17 expression and increased interstitial edema is presented in Figures 5A–D. The number of cells positive for IL-2, IL-5, IL-10, TNF-α, TARC, CD4, and CD8 expression in the lung parenchyma is presented in Table 1 (A). The number of positive cells in the lung parenchyma showed an increase in their contents in the OVA and OVA-LPS groups compared to the SAL group (*p* < 0.05). The OVA-LPS group showed an increase in all markers compared to the OVA group, except for TNF-α and IL-17 (*p* < 0.05). Treatment with anti-IL-17 reduced the levels of inflammatory markers evaluated in the OVA anti-IL-17 and the OVA-LPS anti-IL-17 groups compared to the OVA and OVA-LPS groups, respectively (*p* < 0.05). Regarding interstitial edema, an increase was observed in the OVA and OVA-LPS groups compared to the SAL group (*p* < 0.05). In the OVA-LPS group, an increase was observed in the level of interstitial edema compared to the OVA group (*p* < 0.05). Treatment with anti-IL-17 in the OVA anti-IL-17 and OVA-LPS anti-IL-17 groups decreased the regions of interstitial edema compared to the OVA and OVA-LPS groups (*p* < 0.05).

**Table 1:** Absolute values of inflammatory markers, Treg, and antigen-presenting cells in the pulmonary parenchyma.

	SAL	OVA	OVA anti-IL-17	OVA-LPS	OVA-LPS anti-IL-17
**(A) Inflammatory markers**					
TARC (cells/10^4^ μm^2^)	1.11 ± 0.25	4.32 ± 0.38*	1.17 ± 0.10**	5.61 ± 0.41^*,#^	1.80 ± 0.38**
CD4 (cells/10^4^ μm^2^)	1.87 ± 0.16	4.82 ± 0.51*	1.84 ± 0.32**	6.59 ± 0.64^*,#^	2.40 ± 0.37**
CD8 (cells/10^4^ μm^2^)	0.36 ± 0.11	2.96 ± 0.38*	0.67 ± 0.09**	5.37 ± 0.65^*,#^	1.94 ± 0.30**
IL-2 (cells/10^4^ μm^2^)	0.92 ± 0.08	3.21 ± 0.16*	1.69 ± 0.18**	5.30 ± 0.27^*,#^	1.94 ± 0.10**
IL-5 (cells/10^4^ μm^2^)	0.94 ± 0.19	5.20 ± 0.49*	1.74 ± 0.13**	7.36 ± 0.56^*,#^	2.06 ± 0.34**
IL-10 (cells/10^4^ μm^2^)	0.54 ± 0.09	4.10 ± 0.25*	0.78 ± 0.10**	6.22 ± 0.30^*,#^	1.72 ± 0.18**
TNF-α (cells/10^4^ μm^2^)	1.18 ± 0.37	5.43 ± 0.54*	1.50 ± 0.34**	3.15 ± 0.62*	1.65 ± 0.49**
**(B) Markers Treg and antigen-presenting cell**					
Dendritic cells (cells/10^4^ μm^2^)	0.44 ± 0.10	3.42 ± 0.43*	1.06 ± 0.25**	7.23 ± 0.93^*,#^	2.00 ± 0.26**
FOXP3 (cells/10^4^ μm^2^)	1.37 ± 0.29	8.78 ± 0.74*	5.77 ± 0.61**	11.30 ± 1.32^*,#^	5.71 ± 0.93**

*Inflammatory markers for TARC, CD4, CD8, IL-2, IL-5, IL-10, and TNF-α are presented as positive cells/10^4^ μm^2^, and regulatory T and antigen-presenting cell markers for dendritic cells and FOXP3 are presented as positive cells/10^4^ μm^2^*.

*p < 0.05 in comparison with the SAL group.

**p < 0.05 in comparison with the OVA and OVA-LPS groups.

*^#^p < 0.05 in comparison with the OVA group*.

### Effects of Anti-IL-17 on Regulatory T Cells (Treg) and on Antigen-Presenting Cells (APC)

The numbers of FOXP3 (Treg) and Dendritic cells (APC) in the lung parenchyma is presented in Table 1 (B). There was an increase in the number of positive cells in the lung parenchyma in both the OVA and OVA-LPS groups compared to the SAL group (*p* < 0.05). The OVA-LPS group showed an increase relative to the OVA group (*p* < 0.05). Treatment with anti-IL-17 attenuated all inflammatory markers evaluated in the OVA anti-IL-17 and OVA-LPS anti-IL-17 groups compared to the OVA and OVA-LPS groups (*p* < 0.05).

### Effects of Anti-IL-17 on Extracellular Matrix Remodeling

The number of cells positive for MMP-9, MMP-12, TIMP-1, and TGF-β, as well as the volume fraction of collagen fibers I and III, decorin, actin, biglycan, lumican, and fibronectin in the lung parenchyma, is presented in Table 2. All markers were increased in the OVA and OVA-LPS groups compared to the SAL group (*p* < 0.05). The OVA-LPS group showed an increase in the volume fraction of biglycan, fibronectin, and collagen fibers I and III compared to the OVA group (*p* < 0.05). The numbers of cells positive for MMP-9, MMP-12, and TGF-β were increased compared to the OVA group (*p* < 0.05). There was no increase in the lumican, actin and decorin levels in OVA-LPS animals compared to OVA animals. There was a decrease in all markers in the OVA anti-IL-17 and OVA-LPS anti-IL-17 groups compared to non-treated animals (OVA and OVA-LPS groups, respectively, *p* < 0.05).

**Table 2:** Absolute values for remodeling markers in the pulmonary parenchyma.

Remodeling markers	SAL	OVA	OVA anti-IL-17	OVA-LPS	OVA-LPS anti-IL-17
MMP-9 (cells/10^4^ μm^2^)	2.70 ± 0.46	8.11 ± 0.47*	2.57 ± 0.36**	16.50 ± 0.62^*,#^	9.75 ± 0.43**
MMP-12 (cells/10^4^ μm^2^)	0.32 ± 0.06	7.51 ± 0.48*	1.36 ± 0.26**	11.00 ± 1.96^*,#^	1.62 ± 0.28**
TIMP-1 (cells/10^4^ μm^2^)	1.11 ± 0.18	8.29 ± 0.85*	2.72 ± 0.48**	9.96 ± 0.95*	3.95 ± 0.56**
TGF-β (cells/10^4^ μm^2^)	1.65 ± 0.23	31.60 ± 2.22*	19.84 ± 1.53**	42.06 ± 1.91^*,#^	8.35 ± 0.75**
Collagen Fibers I (%)	12.16 ± 0.88	22.25 ± 2.16*	13.58 ± 1.12**	25.19 ± 1.48	20.08 ± 0.72**
Collagen Fibers III (%)	0.89 ± 0.09	13.47 ± 0.62*	8.17 ± 0.53**	20.10 ± 0.80^*,#^	7.89 ± 0.71**
Decorin (%)	1.62 ± 0.29	52.68 ± 2.60*	2.77 ± 0.43**	55.66 ± 2.37*	8.00 ± 0.92**
Lumican (%)	14.03 ± 0.18	27.39 ± 1.02*	21.64 ± 1.47**	26.57 ± 0.68*	15.33 ± 0.54**
Actin (%)	8.05 ± 1.84	28.44 ± 1.99*	12.80 ± 1.29**	29.90 ± 1.93*	6.9 ± 1.66**
Biglycan (%)	4.13 ± 0.23	12.47 ± 0.71*	5.39 ± 1.08**	20.68 ± 2.83^*,#^	5.00 ± 0.66**
Fibronectin (%)	4.60 ± 0.47	40.27 ± 1.65*	11.17 ± 1.06**	48.56 ± 2.03^*,#^	10.20 ± 0.78**

*Remodeling markers for MMP-9, MMP-12, TIMP-1, and TGF-β are expressed as positive cells/10^4^ μm^2^. Collagen fibers I and II, decorin, lumican, actin, biglycan, and fibronectin are presented as volume fraction expressed as percentages of area (%)*.

*p < 0.05 in comparison with the SAL group.

**p < 0.05 in comparison with the OVA and OVA-LPS groups.

*^#^p < 0.05 in comparison with the OVA group*.

### Effects of Anti-IL-17 on Oxidative Stress

**Figure 6:**
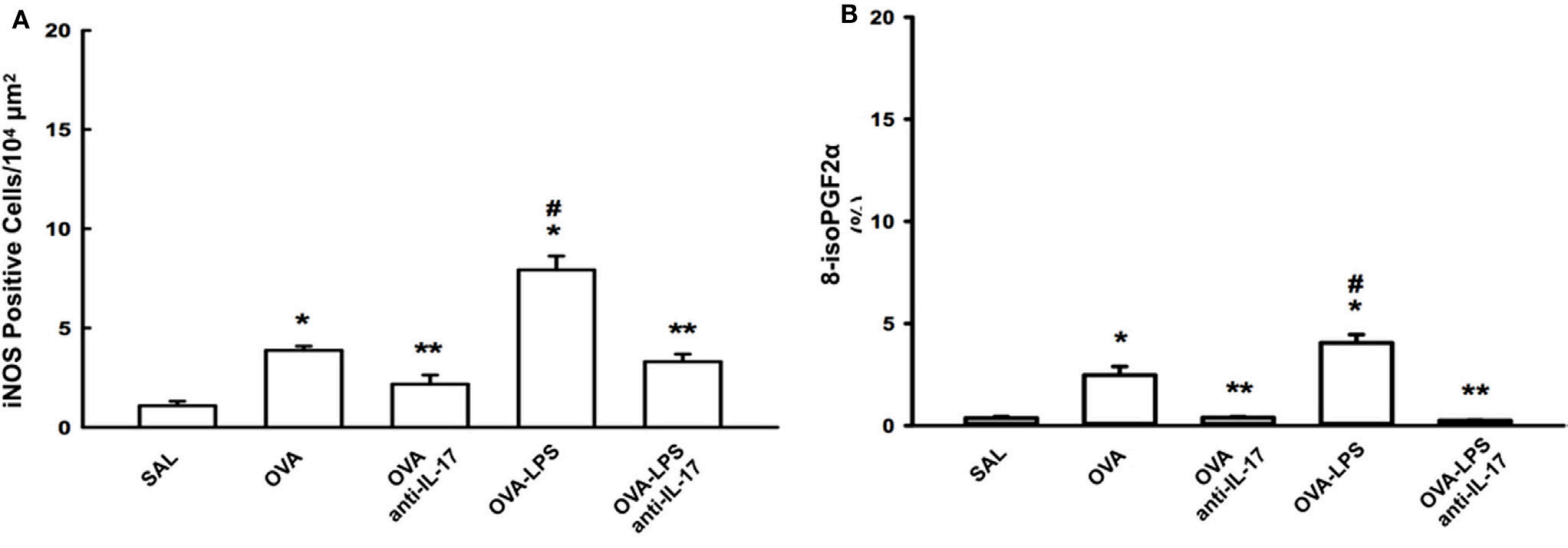
Effects of anti-IL-17 on oxidative stress. **(A,B)**. **(A)** iNOS-positive cells and **(B)** the 8-iso-PGF2α volume fraction. The results are expressed as positive cells/10^4^ µm^2^, and the volume fraction is expressed as percentages of area (%). Data are presented as the means ± SEs. The differences were considered significant when *p* < 0.05 (*p < 0.05 vs. SAL group; **p < 0.05 vs. OVA and OVA-LPS groups; ^#^*p* < 0.05 vs. OVA group).

The numbers of cells positive for iNOS and the volume fractions of PGF-2-α isoprostane in the lung parenchyma are shown in Figures 6A,B. Both markers in the OVA and OVA- LPS groups were increased compared to the SAL group (*p* < 0.05). There was an increase in the OVA-LPS group in the number of cells positive for iNOS and the volume fraction of PGF-2-α isoprostane compared to the OVA group (*p* < 0.05). Treatment with anti-IL-17 attenuated these markers in OVA anti-IL-17 and OVA-LPS anti-IL-17 groups compared to the OVA and OVA-LPS groups (*p* < 0.05).

### Effects of Anti-IL-17 on Signaling Pathways

**Figure 7:**
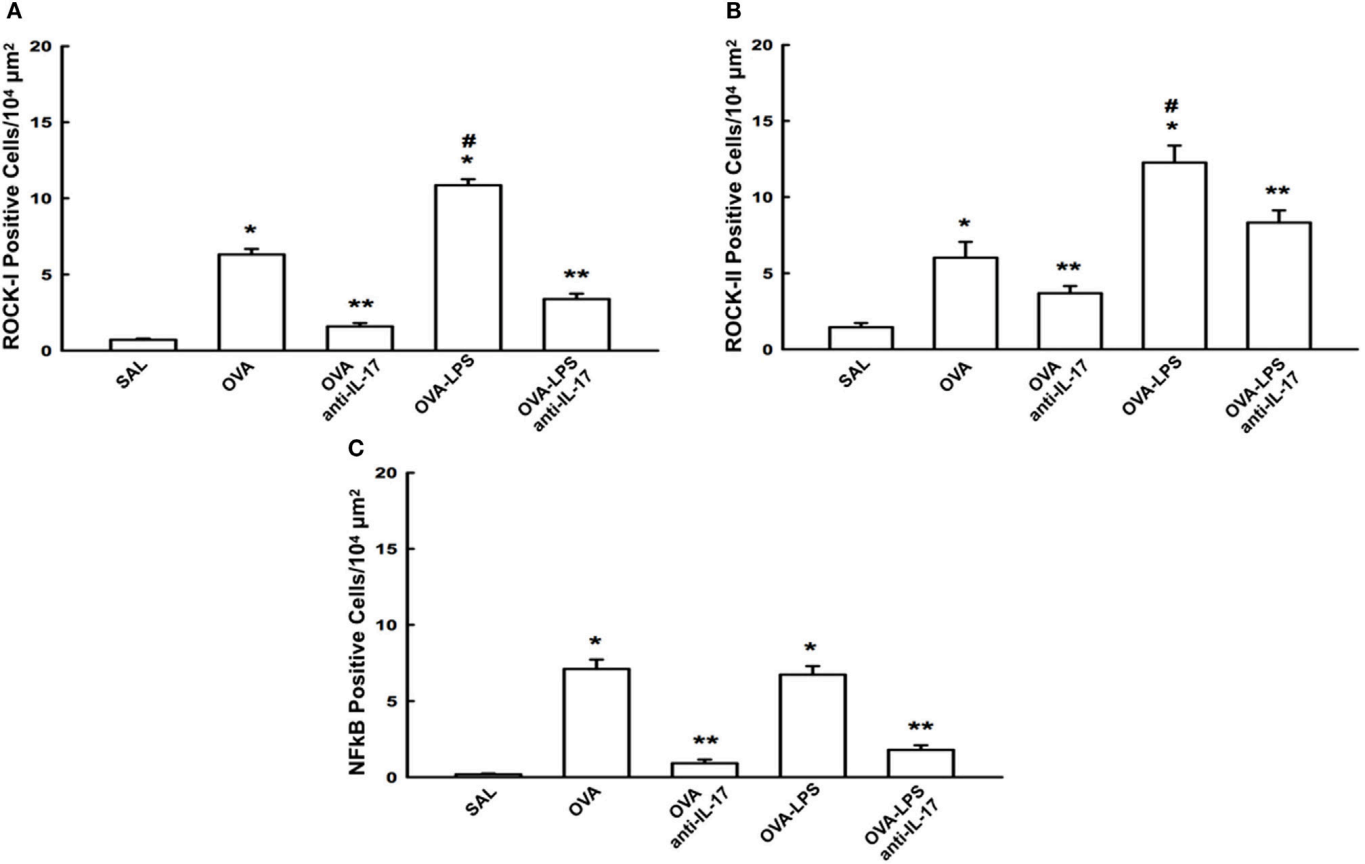
Effects of anti-IL-17 on signaling pathways. **(A–C)**. **(A)** ROCK-1, **(B)** ROCK-2, and **(C)** NF-κB-positive cells. The results are expressed as positive cells/10^4^ µm^2^ Data are presented as the means ± SEs. The differences were considered significant when *p* < 0.05 (*p < 0.05 vs. SAL group; **p < 0.05 vs. OVA and OVA-LPS groups; ^#^*p* < 0.05 vs. OVA group).

The cellular expression levels of NF-κB, ROCK-1, and ROCK-2 in the lung parenchyma are shown in Figures 7A–C. We observed an increase in the numbers of cells positive for these markers in the OVA and OVA-LPS groups compared to the SAL group (*p* < 0.05). There was an increase in the OVA-LPS group in the number of positive cells for ROCK-1 and ROCK-2 compared to the OVA group (*p* < 0.05). In the OVA-LPS group, there was no increase in the number of NF-κB-positive cells. Anti-IL-17 treatment in the OVA anti-IL-17 and OVA-LPS anti-IL-17 group ameliorated all increases in these markers compared to the OVA and OVA-LPS groups, respectively (*p* < 0.05).

### Effects of IL-17 Inhibition Through Qualitative Analysis

**Figure 8:**
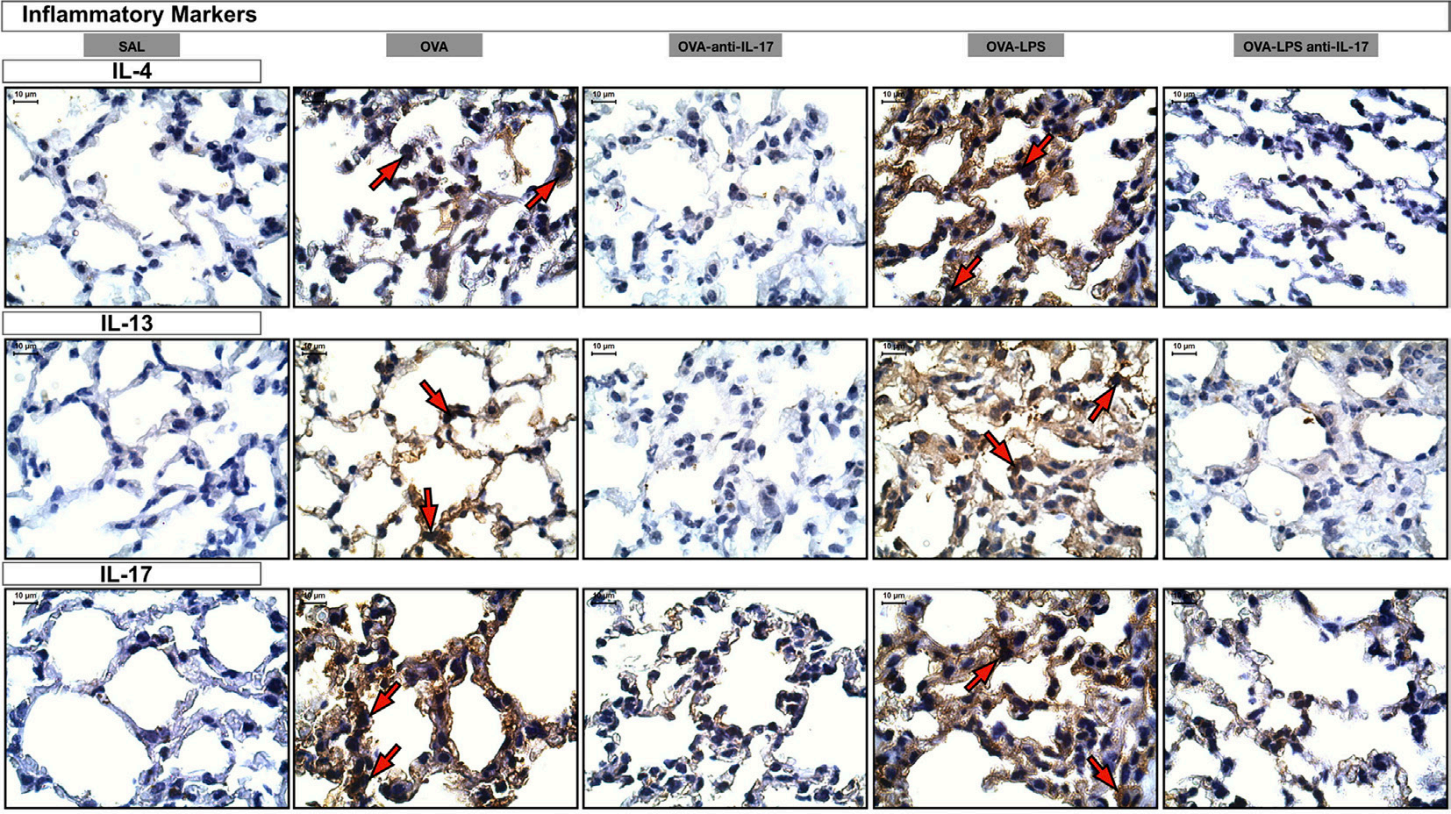
Inflammatory markers. Photomicrographs of extracellular matrix inflammatory features of alveolar walls measuring IL-4, IL-13, and IL-17. The red arrows indicate positive cells for IL-4, IL-13, and IL-17. All images are presented at a magnification of 1,000×, scale bars = 10 µm. The experimental groups are represented as: SAL, OVA, OVA anti-IL-17, OVA-LPS, and OVA-LPS anti-IL-17.

Photomicrographs of extracellular matrix inflammatory features of alveolar walls measuring IL-4, IL-13 and IL-17 are shown in Figure 8. The animals exposed to only ovalbumin and ovalbumin more LPS (OVA and OVA-LPS groups) presented prominent increases in the numbers of positive cells, compared with the control animals (SAL group). Anti-IL-17 treatment in the OVA anti-IL-17 and OVA-LPS anti-IL-17 group ameliorated all increases in these markers.

**Figure 9:**
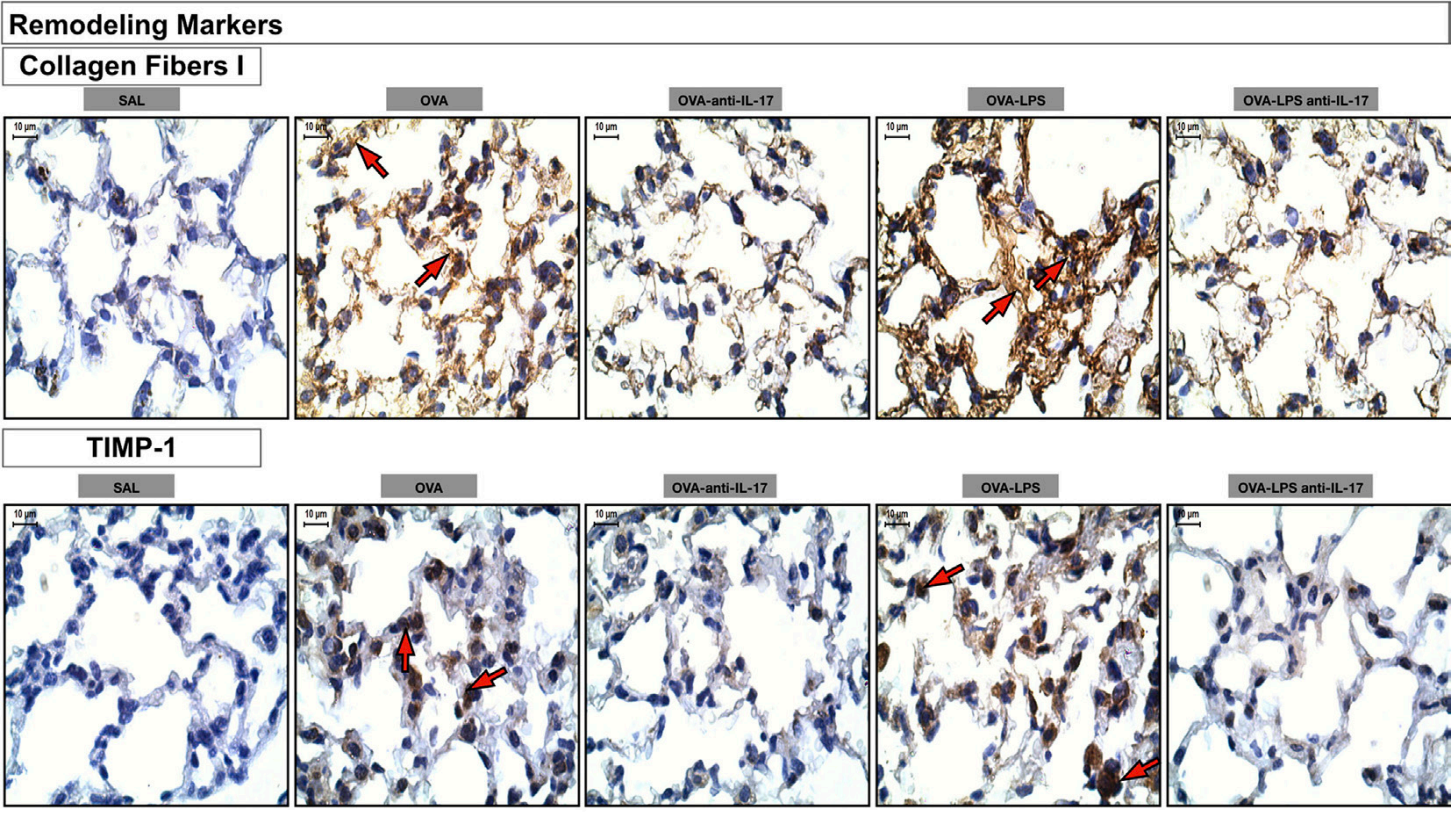
Remodeling markers. Photomicrographs of extracellular matrix remodeling features in the alveolar walls: collagen fibers I and metalloproteinase inhibitor (TIMP-1). The red arrows indicate the collagen fibers and positive cells for TIMP-1. All images are presented at a magnification of 1,000×, scale bars = 10 µm. The experimental groups are represented as: SAL, OVA, OVA anti-IL-17, OVA-LPS, and OVA-LPS anti-IL-17.

Photomicrographs of extracellular matrix remodeling features in the alveolar walls: collagen fibers I and metalloproteinase inhibitor (TIMP-1) is presented in Figure 9. The animals treated with ovalbumin and LPS (OVA, OVA-LPS group) presented increases in volume fraction and number of positive cells compared to the control group (SAL). There was a decrease in all markers in the OVA anti-IL-17 and OVA-LPS anti-IL-17 groups compared to non-treated animals.

**Figure 10:**
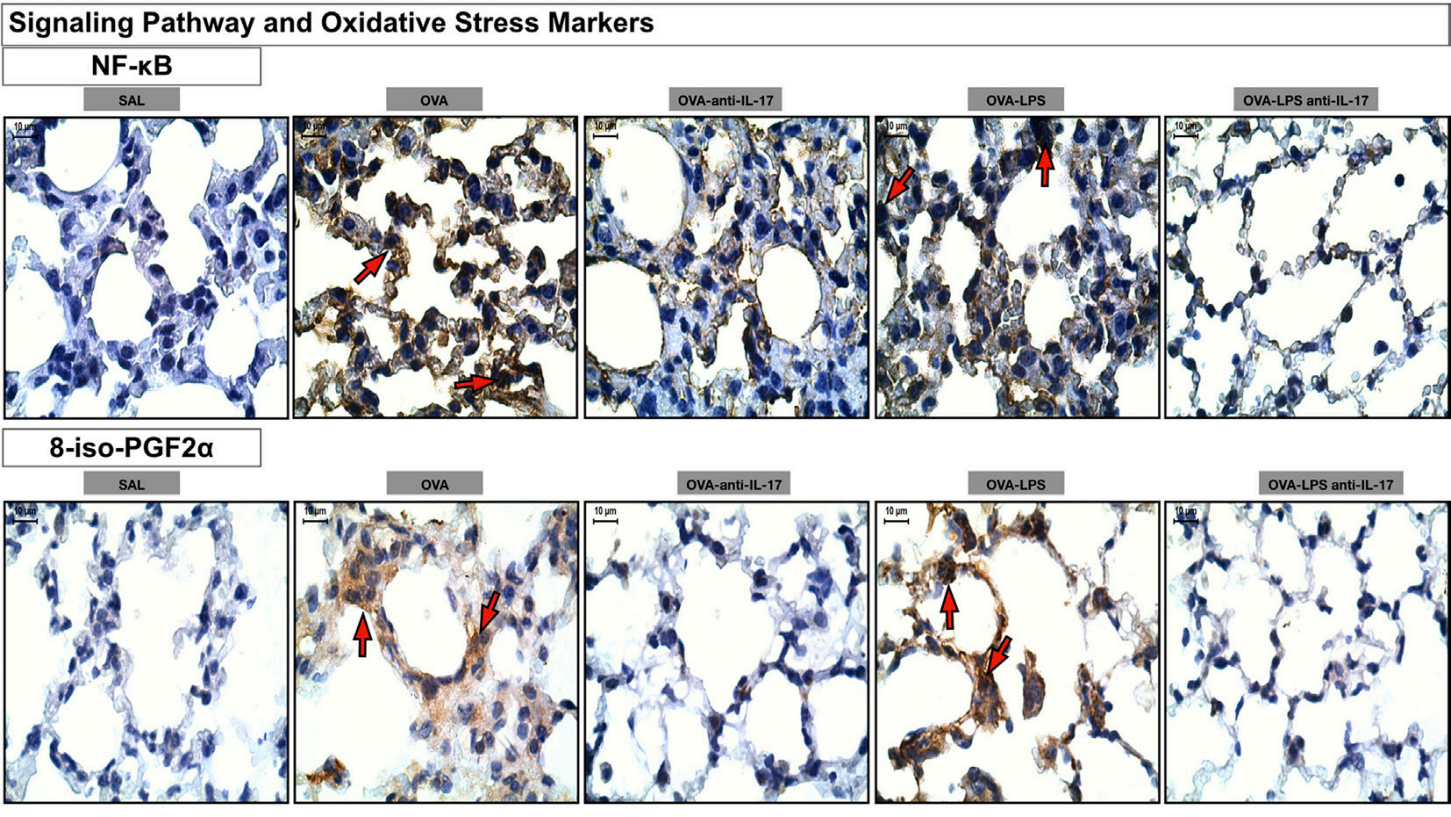
Oxidative stress and Signaling Pathway markers. Photomicrographs of immunohistochemical analyzes of oxidative stress and signaling pathways markers present in the alveolar walls, as represented by NF-κB and 8-iso-PGF2α. The red arrows indicate positive cells for NF-κB and positive area of 8-iso-PGF2α. All images were analyzed at a magnification of 1,000×, scale bars = 10 µm. Experimental groups are represented as: SAL, OVA, OVA anti-IL-17, OVA-LPS, and OVA-LPS anti-IL-17.

Photomicrographs of immunohistochemical analysis of oxidative stress and signaling pathways markers present in the alveolar walls, as represented by NF-κB and 8-iso-PGF2α are shown in Figure 10. There was an increase in the number of cells positive for iNOS and the volume fraction of PGF-2-α iso-prostane in the OVA and OVA-LPS non-treated groups compared to the SAL group. Anti-IL-17 treatment in the OVA anti-IL-17 and OVA-LPS anti-IL-17 group ameliorated all increases in these markers.

## Discussion

We evaluated the effects of inhibition of IL-17 in the present study, using an anti-IL-17 monoclonal antibody, on distal pulmonary parenchyma responses characterized by the infiltration of inflammatory cells, differential release of cytokines and chemokines, oxidative stress, extracellular matrix remodeling, signaling pathways, and IL-6 gene expression in an experimental model of LPS-exacerbated asthma. An attenuation of the inflammatory responses of Th1, Th2, and Th17 cytokines, Treg cells and APCs in both the OVA anti-IL-17 and OVA-LPS anti-IL-17 groups was observed, as represented by reductions in the total numbers of inflammatory cells, macrophages, neutrophils, and eosinophils in the BALF. However, did not there was difference in the numbers of lymphocytes, which we considered that was already expected, since these cells are mainly attached to the airway walls and to the alveolar septum. Several studies showed a low number of lymphocytes compared to eosinophils in BALF in asthma animal models and in asthmatics (2, 31–33). The anti-IL-17 treatment also decreased the markers of extracellular matrix remodeling, reduction in the volume fraction of actin, biglycan, collagen fibers I and III, decorin, lumican and fibronectin, as well as the cellular expression levels of MMP-9, MMP-12, TIMP-1, and TGF-β. Consistently, a decrease in the numbers of cells positive for iNOS and activation of PGF-2-α isoprostane in OVA-anti-IL-17 and OVA-LPS anti-IL-17 groups and similarly, in the signaling pathways mediated by Rho kinases 1 and 2 and NF-κB.

The experimental model utilized here comprised two distinct phases. In the first stage, we induced allergic inflammation through repeated exposures to ovalbumin. At the second stage, our objective was to study an exacerbated asthma model, using a low dose of LPS as previously described (4). We did not consider that this low doses of LPS (0.01 mg/ml) cause acute lung injury based on previous studies in which ALI was induced by doses of LPS at about 50–100 times greater (34, 35). Fodor et al. (35) used increasing doses of LPS (3, 5, and 10 mg/kg) in order to quantify the severity of the dose-dependent effect. They concluded that the findings confirmed sepsis and alveolar–capillary membrane damage in a dose-dependent manner. However, comparing with our work the dose of 3 mg/kg (adjusted to mg per animal) was equivalent to 130 times greater than the one used in our protocol. Reinforcing our hypothesis and in order to rule out the possibility of alveolar damage by this low dose of LPS, we performed the evaluation of alveolar mean (LM) interception (36). LM is an index through which we evaluate the mean diameter of the distal air spaces in order to prove alveolar destruction. We observed that there were no significant differences among all groups.

In previous studies, we demonstrated that this experimental model of allergic inflammation induced by repeated exposures of antigen showed several characteristics present in the pathophysiology of asthma, such as intense constriction, eosinophilic inflammation, oxidative stress, and remodeling of the extracellular matrix in the distal parenchyma (17, 20, 25, 37–39). Concerning the increase of Th1/Th2 and Th17 cytokines observed in the present study, increased levels of CD4+ and CD8+ cells activate immune responses in both humans and experimental mammalian models. Since CD4+ is considered a marker for Th17 cells (40–42), Wang and Wills-Karp (43) demonstrated that the CD4+/Th2 cells induced inflammation during the chronic phase and these cells produce IL-17.

The number of IL-4 and IL-13 positive cells was higher than that observed in the number the of IL-17 positive cells. This increase may be related to an effect of another cytokine. One possibility is the IL-6, since this cytokine has emerged as a regulator of CD4 responses and may induce Th1 and Th2 responses (44). Corroborating with these findings, Diehl et al. (45) showed that IL-6 promotes the production of IL-4 and IL-13 in animal models of asthma. We observed a potentiation of IL-6 in the OVA-LPS group in the same way as IL-4 and IL-13. To confirm the importance of IL-6 as the involved mechanism, we checked these data by PCR and immunohistochemistry. Corroborating with our findings, Bae et al. (14) showed in knockout animals for IL-17A a decrease of neutrophils, IL-6, and IL-17. Nevertheless, IL-13 and IL-17 inhibition protected the animals from eosinophilia, mucus hyperplasia, and hyperresponsiveness of the airways and the elimination of neutrophilic inflammation, suggesting the effectiveness of combination therapies that control both Th2 and Th17 responses (46). Herein, we demonstrated that anti-IL-17 treatment attenuated IL-4, IL-5, and IL-13-positive cell responses in the lung parenchyma in the OVA anti-IL-17 and OVA-LPS anti-IL-17 groups.

The function of Foxp3 is to differentiate and expand Treg cells and is a specific marker for this differentiation (47, 48). After Tregs are activated, they express high levels of TGF-β and IL-10, which play an anti-inflammatory role (49). It is known that in mice, TGF-β and IL-6 cytokines are essential for the differentiation of Th17 cells. Inducing the production of IL-17A and IL-17F (50). In addition, Chakir et al. (51) showed a positive correlation between the severity of the disease and Th17 phenotype in patients with moderate and severe asthma.

Concerning alterations in remodeling of the extracellular matrix in the distal parenchyma, an increase in MMP-9-, MMP-12-, TIMP-1-, TGF-β-positive cells and collagen fibers I and III, decorin, lumican, actin, biblycan, and fibronectin volume fraction in the OVA group compared to SAL. Treatment with anti-IL-17 in the OVA and OVA-LPS animals reduced these responses. It was observed in the OVA-LPS group an increase in the responses of MMP-9, MMP-12, TGF-β, fibronectin, and biglycan.

However, the following markers TIMP-1, decorin, lumican, and actin did not differ between OVA and OVA-LPS group. It had been previously shown that LPS in cell cultures an increase in decorin (RNA protein) after 24 h. Nonetheless, the major effect was at 48 h (52). We used only one dose of anti-IL17 after LPS administration. One possible explanation is that only one dose was not sufficient to control the stimuli for decorin production induced by LPS. Analyzing lumican responses, we observed that OVA and OVA-LPS groups were not different. However, anti-IL-17 contributed for a greater reduction of lumican in these groups. Since we observed the same response in the TGF-β assessment, we hypothesized that the reduction of TGF-β by anti IL-17 contributed to the effects observed in the lumican response. These data are in accordance with Krishnan et al. (53), which showed that TGF-β increases lumican expression. In relation to actin, the anti-IL-17 treatment showed a tendency to decrease actin in the OVA-LPS anti IL-17 group. Qin et al. (54) showed that TGF-β was also able to increase actin expression in allergic inflammation models.

In the current study, we found that anti-IL-17 therapy neutralized the remodeling of the lung parenchyma, and we quantified the volume fraction of collagen fibers I and III. We observed that anti-IL-17 treatment mitigated the deposition of collagen fibers in the ECM. These results suggest that anti-IL-17 therapy not only controls inflammation but also remodeling process.

The results of the present study showed a high number of cells positive for MMP-9 and MMP-12 in the OVA-LPS group. Several inflammatory cytokines, such as TNF-a and the antigenic agent LPS, stimulate the synthesis of MMPs in neutrophils (55), and LPS stimulates neutrophil recruitment (56, 57). Oshita et al. (58) had shown that MMP-9 and MMP-12 represent markers of inflammation and remodeling. In addition to the remodeling process, these MMPs also participate in the inflammatory process through the modulation of signaling Th2 cells or through the regulation of leukocyte infiltration in the tissues of the distal parenchyma (59).

Moreover, the reduction of oxidative stress and signaling pathways in response to treatment with anti-IL-17 may have contributed to the reduction of ECM remodeling. We observed an increase in the volume fraction of PGF2-α, iNOS and cells positive for ROCK-1, ROCK-2, and NF-κB. These markers were reduced after treatment with anti-IL-17.

Righetti et al. (20) and Possa et al. (25) showed that inhibition of ROCK-1 and ROCK-2 contributes to the reduction of eosinophilic recruitment, hyperresponsiveness, and markers of the remodeling process in an asthma model. These findings had positive correlations with the functional responses and the markers of inflammation and remodeling. These studies corroborate our results, suggesting that anti-IL-17 was able to counteract this signaling pathway.

Consistent with these results, Prado et al. (60) demonstrated that treatment with an iNOS inhibitor reduced MMP-9, TIMP-1, and TGF-β levels in the airways in an asthma model. These mediators modulate the production of the collagen and elastic fibers, contributing to the remodeling of the ECM. Starling et al. (37) also showed a significant reduction of PGF2-α isoprostane in animals sensitized with ovalbumin and treated with specific iNOS inhibitors.

Transcription factors are involved in all these processes, including NF-κB, which is considered a critical modulator of inflammation in the pathogenesis of lung diseases (61). In an attempt to determine the importance of NF-κB during sensitization to the antigen in the murine asthma model, Pantano et al. (62) demonstrated that the epithelial activation of NF-κB promoted neutrophilia and eosinophilia and increased the levels of IL-17 and IL-4. Notably, the activation of this pathway might also increase the levels of NOS and arginase (38, 63).

Our study has certain limitations. We used a monoclonal antibody to IL-17 in an experimental animal model, and we cannot directly extrapolate these findings to those expected in humans, although there are several ongoing clinical tests for severe asthmatic patients. Regarding the remodeling process, we evaluated the alterations immediately after the end of the protocol. It would be interesting to evaluate if these alterations were maintained after weeks or months of the end of the experiment. In addition, the confirmation of all alterations observed by immunohistochemistry, using another methodology, such as ELISA could be performed in future studies. In the present one, we did it only for IL-6 considering the regulatory importance of this cytokine on CD4 responses, as previously discussed. However, our study has many strengths since we showed the importance of investigating the Th17 profile in chronic allergic inflammation exacerbated by LPS. We demonstrated a potential of anti-IL17 to attenuate and/or control inflammatory responses, airway remodeling, oxidative stress pathways, as well as the mechanisms involved in the activation of NF-κB and ROCK-1 and 2 in the lungs with allergic inflammation even though there was a second inflammatory stimulus induced by the exacerbation. Further studies should, therefore, be performed to reveal other pathways that lead to these changes.

## Conclusion

In this study, it was demonstrated that inhibition of IL-17 contributes to the control of Th1/Th2/Th17-mediated inflammation, Treg cells, APCs, chemokine expression, extracellular matrix remodeling, and oxidative stress in the lung parenchyma in a model of chronic allergic inflammation exacerbated by LPS. This treatment represents a promising therapeutic strategy, although further studies are necessary.

## Ethics Statement

The present study was approved by the Research Ethics Committee of the Hospital das Clínicas of the Faculty of Medicine of the University of São Paulo (Case No. 141/16). This protocol was repeated twice and used a total of 60 animals male BALB/c mice from the Medical School at the University of São Paulo were utilized in accordance with the Guideline to Care and Use of Laboratory Animals, published by the National Institutes of Health (NIH publication 85-23, revised in 1985). On average, the body weight of the animals was approximately 20–25 g at the beginning of the sensitization protocol.

## Author Contributions

IT, MM, RR, and LC conceived the study. LC, RR, LA, TS, and FS performed the experiments. LC, RR, LA, TS, FS, and SF analyzed the data. IT, RR, MM, CP, MC, MA-V and BS-R contributed reagents/materials/analysis tools. IT, RR, EL, and LC wrote the paper.

## Conflict of Interest Statement

The authors declare that the research was conducted in the absence of any commercial or financial relationships that could be construed as a potential conflict of interest.

## References

[1] Dougherty R Fahy JV. Acute exacerbations of asthma: epidemiology, biology and the exacerbation-prone phenotype. Clin Exp Allergy (2009) 39(2):193–202. 10.1111/j.1365-2222.2008.03157.x 19187331 PMC2730743

[2] GINA. Global Strategy for Asthma Management and Prevention. Global Initiative for Asthma (GINA) (2015). Available from: http://www.ginaasthma.org

[3] Petroni RC Biselli PJ de Lima TM Theobaldo MC Caldini ET Pimentel RN Hypertonic saline (NaCl 7.5%) reduces LPS-induced acute lung injury in rats. Inflammation (2015) 38(6):2026–35. 10.1007/s10753-015-0183-4 25962375

[4] Starkhammar M Kumlien Georén S Swedin L Dahlén SE Adner M Cardell LO. Intranasal administration of poly(I:C) and LPS in BALB/c mice induces airway hyperresponsiveness and inflammation via different pathways. PLoS One (2012) 7(2):e32110. 10.1371/journal.pone.0032110 22355412 PMC3280225

[5] Venancio TM Machado RM Castoldi A Amano MT Nunes VS Quintao ECR CETP lowers TLR4 expression which attenuates the inflammatory response induced by LPS and polymicrobial sepsis. Mediators Inflamm (2016) 2016:1784014 10.1155/2016/1784014 27293313 PMC4880711

[6] Roussel L Houle F Chan C Yao Y Bérubé J Olivenstein R IL-17 promotes MAPK dependent endothelial activation enhancing neutrophil recruitment to sites of inflammation. J Immunol (2010) 184(8):4531–7. 10.4049/jimmunol.0903162 20228195

[7] Fajt ML Wenzel SE Development of new therapies for severe asthma. Allergy Asthma Immunol Res (2016) 9(1):3–14. 10.4168/aair.2017.9.1.3 PMC5102833 27826957

[8] Zhao Y Yang J Gao YD. Altered expressions of helper T cell (Th)1, Th2, and Th17 cytokines in CD8(+) and γδ T cells in patients with allergic asthma. J Asthma (2011) 48(5):429–36. 10.3109/02770903.2011.570403 21492057

[9] Al-Muhsen S Letuve S Vazquez-Tello A Pureza MA Al-Jahdali H Bahammam AS Th17 cytokines induce profibrotic cytokines release from human eosinophils. Respir Res (2013) 14:34 10.1186/1465-9921-14-34 23496774 PMC3602055

[10] Pigati PA Righetti RF Possa SS Romanholo BS Rodrigues AP dos Santos AS Y-27632 is associated with corticosteroid-potentiated control of pulmonary remodeling and inflammation in guinea pigs with chronic allergic inflammation. BMC Pulm Med (2015) 15:85. 10.1186/s12890-015-0073-4 26264367 PMC4531528

[11] Kudo M Melton AC Chen C Engler MB Huang KE Ren X IL-17A produced by ab T cells drives airway hyper-responsiveness in mice and enhances mouse and human airway smooth muscle contraction. Nat Med (2012) 18(4):547–54. 10.1038/nm.2684 22388091 PMC3321096

[12] Willis CR Siegel L Leith A Mohn D Escobar S Wannberg S IL-17RA signaling in airway inflammation and bronchial hyperreactivity in allergic asthma. Am J Respir Cell Mol Biol (2015) 53(6):810–21. 10.1165/rcmb.2015-0038OC 25919006

[13] Lowe AP Thomas RS Nials AT Kidd EJ Broadley KJ Ford WR LPS exacerbates functional and inflammatory responses to ovalbumin and decreases sensitivity to inhaled fluticasone propionate in a guinea pig model of asthma. Br J Pharmacol (2015) 172(10):2588–603. 10.1111/bph.13080 25586266 PMC4409909

[14] Bae JS Kim JH Kim EH Mo JH. The role of IL-17 in a lipopolysaccharide-induced rhinitis model. Allergy Asthma Immunol Res (2017) 9(2):169–76. 10.4168/aair.2017.9.2.169 28102062 PMC5266111

[15] Dolhnikoff M da Silva LF de Araujo BB Gomes HA Fernezlian S Mulder A The outer wall of small airways is a major site of remodeling in fatal asthma. J Allergy Clin Immunol (2009) 123(5):1090–7, 1097.e1. 10.1016/j.jaci.2009.02.032 19361849

[16] Tulic MK Hamid Q Contribution of the distal lung to the pathologic and physiologic changes in asthma: potential therapeutic target Roger S. Mitchell lecture. Chest (2003) 123(3 Suppl):348S–55S. 10.1378/chest.123.3_suppl.348S 12628971

[17] Angeli P Prado CM Xisto DG Silva PL Pássaro CP Nakazato HD Effects of chronic L-NAME treatment lung tissue mechanics, eosinophilic and extracellular matrix responses induced by chronic pulmonary inflammation. Am J Physiol Lung Cell Mol Physiol (2008) 294(6):L1197–205. 10.1152/ajplung.00199.2007 18359886

[18] Benayoun L Druilhe A Dombret MC Aubier M Pretolani M. Airway structural alterations selectively associated with severe asthma. Am J Respir Crit Care Med (2003) 167(10):1360–8. 10.1164/rccm.200209-1030OC 12531777

[19] Mauad T Ferreira DS Costa MB Araujo BB Silva LF Martins MA Characterization of autopsy-proven fatal asthma patients in São Paulo, Brazil. Rev Panam Salud Publica (2008) 23(6):418–23. 10.1590/S1020-49892008000600007 18644210

[20] Righetti RF Pigati PA Possa SS Habrum FC Xisto DG Antunes MA Effects of Rho-kinase inhibition in lung tissue with chronic inflammation. Respir Physiol Neurobiol (2014) 192:134–46. 10.1016/j.resp.2013.12.012 24373838

[21] National Institutes of Health. Health Research Extension Act of 1985, Public Law 99-158, November 20, 1985. “Animals in Research” U.S. Government Principles for the Utilization and Care of Vertebrate Animals Used in Testing, Research, and Training Public Health Service Policy on Humane Care and Use of Laboratory Animals. (1985). Available from: http://grants.nih.gov/grants/olaw/references/phspol.htm#USGovPrinciples

[22] Arantes-Costa FM Lopes FD Toledo AC Magliarelli-Filho PA Moriya HT Carvalho-Oliveira R Effects of residual oil fly ash (ROFA) in mice with chronic allergic pulmonary inflammation. Toxicol Pathol (2008) 36(5):680–6. 10.1177/0192623308317427 18477768

[23] Barlow JL Flynn RJ Ballantyne SJ McKenzie AN. Reciprocal expression of IL-25 and IL-17A is important for allergic airways hyperreactivity. Clin Exp Allergy (2011) 41(10):1447–55. 10.1111/j.1365-2222.2011.03806.x 21722219

[24] Margraf LR Tomashefski JF Jr Bruce MC Dahms BB. Morphometric analysis of the lung in bronchopulmonary dysplasia. Am Rev Respir Dis (1991) 143(2):391–400. 10.1164/ajrccm/143.2.391 1990959

[25] Possa SS Charafeddine HT Righetti RF da Silva PA Almeida-Reis R Saraiva-Romanholo BM Rho-kinase inhibition attenuates airway responsiveness, inflammation, matrix remodeling, and oxidative stress activation induced by chronic inflammation. Am J Physiol Lung Cell Mol Physiol (2012) 303(11):L939–52. 10.1152/ajplung.00034.2012 23002076

[26] Rocco PR Momesso DP Figueira RC Ferreira HC Cadete RA Légora-Machado A Therapeutic potential of a new phosphodiesterase inhibitor in acute lung injury. Eur Respir J (2003) 22(1):20–7. 10.1183/09031936.03.00108603 12882446

[27] Nakashima AS Prado CM Lancas T Ruiz VC Kasahara DI Leick-Maldonado EA Oral tolerance attenuates changes in in vitro lung tissue mechanics and extracellular matrix remodeling induced by chronic allergic inflammationin guinea pigs. J Appl Physiol (2008) 104(6):1778–85. 10.1152/japplphysiol.00830.2007 18388250

[28] Kiss T Silva PL Huhle R Moraes L Santos RS Felix NS Comparison of different degrees of variability in tidal volume to prevent deterioration of respiratory system elastance in experimental acute lung inflammation. Br J Anaesth (2016) 116(5):708–15. 10.1093/bja/aew093 27106975

[29] Santos CL Santos RS Moraes L Samary CS Felix NS Silva JD Effects of pressure support and pressure-controlled ventilation on lung damage in a model of mild extrapulmonary acute lung injury with intra-abdominal hypertension. PLoS One (2017) 12(5):e0178207. 10.1371/journal.pone.0178207 28542443 PMC5444773

[30] Pinheiro NM Miranda CJ Perini A Câmara NO Costa SK Alonso-Vale MI Pulmonary inflammation is regulated by the levels of the vesicular acetylcholine transporter. PLoS One (2015) 10(3):e0120441. 10.1371/journal.pone.0120441 25816137 PMC4376856

[31] Seitzman GD Sonstein J Kim S Choy W Curtis JL Lung lymphocytes proliferate minimally in the murine pulmonary immune response to intratracheal sheep erythrocytes. Am J Respir Cell Mol Biol (1998) 18(6):800–12. 10.1165/ajrcmb.18.6.3063 9618385 PMC4123639

[32] Tibério IF Turco GM Leick-Maldonado EA Sakae RS Paiva SO do Patrocínio M Effects of neurokinin depletion on airway inflammation induced by chronic antigen exposure. Am J Respir Crit Care Med (1997) 155(5):1739–47. 10.1164/ajrccm.155.5.9154886 9154886

[33] Murakami D Yamada H Yajima T Masuda A Komune S Yoshikai Y Lipopolysaccharide inhalation exacerbates allergic airway inflammation by activating mast cells and promoting Th2 responses. Clin Exp Allergy (2007) 37(3):339–47. 10.1111/j.1365-2222.2006.02633.x 17359384

[34] Abdelmageed ME El-Awady MS Abdelrahim M Suddek GM LPS-RS attenuation of lipopolysaccharide-induced acute lung injury involves NF-κB inhibition. Can J Physiol Pharmacol (2015) 94(2):140–6. 10.1139/cjpp-2015-0219 26544923

[35] Fodor RŞ Georgescu AM Cioc AD Grigorescu BL Cotoi OS Fodor P Time- and dose-dependent severity of lung injury in a rat model of sepsis. Rom J Morphol Embryol (2015) 56(4):1329–37. 26743278

[36] Menden HL Xia S Mabry SM Navarro A Nyp MF Sampath V Nicotinamide adenine dinucleotide phosphate oxidase 2 regulates LPS-induced inflammation and alveolar remodeling in the developing lung. Am J Respir Cell Mol Biol (2016) 55(6):767–78. 10.1165/rcmb.2016-0006OC 27438994 PMC5248953

[37] Starling CM Prado CM Leick-Maldonado EA Lanças T Reis FG Aristóteles LR Inducible nitric oxide synthase inhibition attenuates lung tissue responsiveness and remodeling in a model of chronic pulmonary inflammation in guinea pigs. Respir Physiol Neurobiol (2009) 165(2–3):185–94. 10.1016/j.resp.2008.11.011 19118648

[38] Aristoteles LR Righetti RF Pinheiro NM Franco RB Starling CM da Silva JC Modulation of the oscillatory mechanics of lung tissue and the oxidative stress response induced by arginase inhibition in a chronic allergic inflammation model. BMC Pulm Med (2013) 13:52. 10.1186/1471-2466-13-52 23947680 PMC3751598

[39] Sakoda CP de Toledo AC Perini A Pinheiro NM Hiyane MI Grecco Sdos S Sakuranetin reverses vascular peribronchial and lung parenchyma remodeling in a murine model of chronic allergic pulmonary inflammation. Acta Histochem (2016) 118(6):615–24. 10.1016/j.acthis.2016.07.001 27425653

[40] Galli SJ Tsai M Piliponsky AM. The development of allergic inflammation. Nature (2008) 454(7203):445–54. 10.1038/nature07204 18650915 PMC3573758

[41] Taher YA Henricks PA Oosterhout AJ Allergen-specific subcutaneous immunotherapy in allergic asthma: immunologic mechanisms and improvement. Libyan J Med (2010) 5(1):5303 10.3402/ljm.v5i0.5303 PMC3071166 21483568

[42] Newcomb DC Peebles RS Jr. Th17-mediated inflammation in asthma. Curr Opin Immunol (2013) 25(6):755–60. 10.1016/j.coi.2013.08.002 24035139 PMC3855890

[43] Wang YH Wills-Karp M. The potential role of interleukin-17 in severe asthma. Curr Allergy Asthma Rep (2011) 11(5):388–94. 10.1007/s11882-011-0210-y 21773747 PMC4115366

[44] Rincon M Irvin CG. Role of IL-6 in asthma and other inflammatory pulmonary diseases. Int J Biol Sci (2012) 8(9):1281–90. 10.7150/ijbs.4874 23136556 PMC3491451

[45] Diehl S Chow CW Weiss L Palmetshofer A Twardzik T Rounds L Induction of NFATc2 expression by interleukin 6 promotes T helper type 2 differentiation. J Exp Med (2002) 196(1):39–49. 10.1084/jem.20020026 12093869 PMC2194007

[46] Wakashin H Hirose K Maezawa Y Kagami S Suto A Watanabe N IL-23 and Th17 cells enhance Th2-cell-mediated eosinophilic airway inflammation in mice. Am J Respir Crit Care Med (2008) 178(10):1023–32. 10.1164/rccm.200801-086OC 18787221

[47] Pyzik M Piccirillo CA TGF-beta1 modulates Foxp3 expression and regulatory activity in distinct CD4+ T cell subsets. J Leukoc Biol (2007) 82:335–46. 10.1189/jlb.1006644 17475784

[48] Palomares O Ruckert B Jartti T Kucuksezer UC Puhakka T Gomez E Induction and maintenance of allergen-specific FOXP3? Treg cells in human tonsils as potential first-line organs of oral tolerance. J Allergy Clin Immunol (2012) 129:510–20, 520.e1–9. 10.1016/j.jaci.2011.09.031 22051696

[49] Durrant DM Metzger DW. Emerging roles of T helper subsets in the pathogenesis of asthma. Immunol Invest (2010) 39(4–5):526–49. 10.3109/08820131003615498 20450290 PMC3787690

[50] Korn T Bettelli E Oukka M Kuchroo VK. IL-17 and Th17 cells. Annu Rev Immunol (2009) 27:485–517. 10.1146/annurev.immunol.021908.132710 19132915

[51] Chakir J Shannon J Molet SS Fukakusa M Elias J Laviolette M Airway remodeling-associated mediators in moderate to severe asthma: effect of steroids on TGF-beta, IL-11, IL-17, and type I and type III collagen expression. J Allergy Clin Immunol (2003) 111(6):1293–8. 10.1067/mai.2003.1557 12789232

[52] He W Qu T Yu Q Wang Z Wang H Zhang J Lipopolysaccharide enhances decorin expression through the Toll-like receptor 4, myeloid differentiating factor 88, nuclear factor-kappa B, and mitogen-activated protein kinase pathways in odontoblast cells. J Endod (2012) 38(4):464–9. 10.1016/j.joen.2011.12.021 22414830

[53] Krishnan A Li X Kao WY Viker K Butters K Masuoka H Lumican, an extracellular matrix proteoglycan, is a novel requisite for hepatic fibrosis. Lab Invest (2012) 92(12):1712–25. 10.1038/labinvest.2012.121 23007134 PMC3810270

[54] Qin XJ Zhang GS Zhang X Qiu ZW Wang PL Li YW Protein tyrosine phosphatase SHP2 regulates TGF-β1 production in airway epithelia and asthmatic airway remodeling in mice. Allergy (2012) 67(12):1547–56. 10.1111/all.12048 23057634 PMC3942166

[55] Overbeek SA Braber S Koelink PJ Henricks PA Mortaz E LoTam Loi AT Cigarette smoke-induced collagen destruction; key to chronic neutrophilic airway inflammation? PLoS One (2013) 8(1):e55612 10.1371/journal.pone.0055612 23383243 PMC3561332

[56] Veldhoen M Hocking RJ Atkins CJ Locksley RM Stockinger B. TGFbeta in the context of an inflammatory cytokine milieu supports de novo differentiation of IL-17-producing T cells. Immunity (2006) 24(2):179–89. 10.1016/j.immuni.2006.01.001 16473830

[57] Wilson NJ Boniface K Chan JR McKenzie BS Blumenschein WM Mattson JD Development, cytokine profile and function of human interleukin 17-producing helper T cells. Nat Immunol (2007) 8(9):950–7. 10.1038/ni1497 17676044

[58] Oshita Y Koga T Kamimura T Matsuo K Rikimaru T Aizawa H. Increased circulating 92 kDa matrix metalloproteinase (MMP-9) activity in exacerbations of asthma. Thorax (2003) 58(9):757–60. 10.1136/thorax.58.9.757 12947131 PMC1746799

[59] Ingram JL Kraft M Metalloproteinases as modulators of allergic asthma: therapeutic perspectives. Metalloproteinases Med (2015) 2:61–74. 10.2147/MNM.S63614

[60] Prado CM Yano L Rocha G Starling CM Capelozzi VL Leick-Maldonado EA Effects of inducible nitric oxide synthase inhibition in bronchial vascular remodeling-induced by chronic allergic pulmonary inflammation. Exp Lung Res (2011) 37(5):259–68. 10.3109/01902148.2010.538289 21585312

[61] Gosens R Schaafsma D Bromhaar MMG Vrugt B Zaagsma J Meurs H Growth factor-induced contraction of human bronchial smooth muscle is Rho-kinase-dependent. Eur J Pharmacol (2004) 494(1):73–6. 10.1016/j.ejphar.2004.04.035 15194453

[62] Pantano C Ather JL Alcorn JF Poynter ME Brown AL Guala AS Nuclear factor-kB activation in airway epithelium induces inflammation and hyperresponsiveness. Am J Respir Crit Care Med (2008) 177(9):959–69. 10.1164/rccm.200707-1096OC 18263801 PMC2361423

[63] Ckless K Vliet A Janssen-Heininger Y. Oxidative-nitrosative stress and post-translational protein modifications: implications to lung structure-function relations. Arginase modulates NF-kappaB activity via a nitric oxide-dependent mechanism. Am J Respir Cell Mol Biol (2007) 36(6):645–53. 10.1165/rcmb.2006-0329SM 17218616 PMC1899343

